# 3D Cardiac Cell Culture on Nanofiber Bundle Substrates for the Investigation of Cell Morphology and Contraction

**DOI:** 10.3390/mi8050147

**Published:** 2017-05-05

**Authors:** Xia Liu, Sixing Xu, Xuanlin Kuang, Xiaohong Wang

**Affiliations:** Tsinghua National Laboratory for Information Science and Technology, Institute of Microelectronics, Tsinghua University, Beijing 100084, China; bobo20074447@126.com (X.L.); 6029xusixing@163.com (S.X.); wo382408000@hotmail.com (X.K.)

**Keywords:** three-dimensional cell culture, cardiomyocyte, nanofiber bundles, nanofiber pattern, cell morphology, cell contraction

## Abstract

Cardiac failure is a quite severe condition that can result in life-threatening consequences. Cardiac tissue engineering is thought to be one of the most promising technologies to reconstruct damaged cardiac muscles and facilitate myocardial tissue regeneration. We report a new nanofiber bundle substrate for three-dimensional (3D) cardiac cell culture as a platform to investigate cell morphology and contraction. Polymeric nanofiber bundles with various patterns act as physical cues to align the cardiac cell sheets. Comparing the uniaxial alignment with the randomly distributed pattern, we found that the bundles with the former pattern have more “grooves” for the settlement of cardiomyocytes in a 3D structure than the latter. The cardiomyocytes loaded on the aligned nanofiber bundles tend to grow along the fiber axis. The interfacial structure between a single cardiomyocyte in the cardiac cell sheet and the attached nanofibers was observed using environmental scanning electron microscope. Immunofluorescence imaging showed that the uniaxially aligned nanofibers greatly promoted cell attachment and alignment of the cardiomyocytes because of the matching morphology between the nanofiber pattern and the biological components. Moreover, we concluded that the aligned polymeric nanofibers could be a promising substrate suitable for the anisotropic contraction of cardiac cell sheets.

## 1. Introduction

Cardiac failure is a quite severe condition that can result in life-threatening consequences. Due to the limited number of organ donors, cardiac tissue engineering is thought to be one of the most promising technologies to reconstruct damaged cardiac muscles and facilitate myocardial tissue regeneration. There is a significant need for three-dimensional (3D) matrices to efficiently deliver cardiac cells [1,2]. For many cell lines, the two-dimensional (2D) to 3D matrix transition can induce changes in the expression of some biomarkers [3,4]. 3D cell culture models aim to restore the 3D architecture that characterizes normal tissues [5] and behaves as a surrounding extracellular matrix (ECM) for biomedical sensing and observation [6]. Studies have shown that 3D cell culture platforms can be established by culturing primary cells or cell lines within ECM gels, rotary cell culture systems, and biomaterial scaffolds on low-adherent culture plastics [7,8,9,10,11,12]. For example, the design of porous materials enables new material properties and applications. In particular, because functionalized 3D biomaterials facilitate the studies of engineered tissues under the effect of biochemical stimulants, the development of synthetic 3D ECM biomaterials has become a wide research area. Advanced biomaterials are capable of monitoring cell functionalities throughout the 3D ECM [13,14]. Bioprinting is a widely-used method to build engineered cardiac constructs that resemble native tissue across the macro- to nanoscale [15,16,17,18]. Bioprinting can potentially print alternating heterogeneous cells and tissues, and resemble a vasculature network. Besides, cardiomyocyte adhesion, alignment, organization, and maturation contribute most to cardiac tissue engineering. Specifically, the alignment of elongated cardiomyocytes and their surrounding ECM as well as the distribution of cell-cell junctions have profound implications on the electromechanical interaction of cardiac muscle [19]. The cell culture substrates are crucial cues, as they affect cell morphology and contraction. But less is known about the alignment and morphology of cardiac cell sheets cultured on 3D substrates with customized patterns.

Electrospinning is a simple and versatile technology for the fabrication of various biocompatible micro-/nanofibers for applications in tissue engineering. Compared to other methods, such as electrohydrodynamics and bioprinting, the electrospinning technique has outstanding advantages in nanoscale manipulation and patterning, especially for the study of cell patterning and morphology [20,21]. Electrospun fibers have several advantages which are very critical for their application, including high specific surface area for better nutrition perfusion and cell interconnection, controlled fiber diameters to adapt to fibrous architectures, and the ability to incorporate bioactive ingredients into their polymers for tunable properties [22]. Electrospinning conditions, such as polymer concentration, humidity, solvent mixture, direct current (DC) voltage and airflow, have an effect on the fiber characteristics in terms of morphology and crystallinity [23,24]. In addition, the fiber pattern is dependent on the pattern of the collector. Electrospinning can yield various patterned fibers, such as random or aligned patterns, which greatly influence cell morphology and function, especially in muscle and nerve tissue engineering [25,26,27,28]. For example, uniaxially aligned nanofibers provide better orientation of cardiac cells, and can act as potential scaffolds for cardiac tissue reconstruction [29]. 

Here, we provide a detailed insight into 3D cardiac cell culture on nanofiber bundle substrates for the investigation of cell morphology and contraction. To determine the underlying mechanisms of the interactions of the nanofiber bundle substrate with the cardiomyocyte functionality, we examine the interfacial structure of the cardiomyocytes and nanofibers, as well as the internal structure and the contraction force of the cardiomyocytes. The fundamental investigation at the protein level of the cardiomyocytes on the nanofibers establishes their potential as a 3D cell culture scaffold.

## 2. Methodology

**Figure 1:**
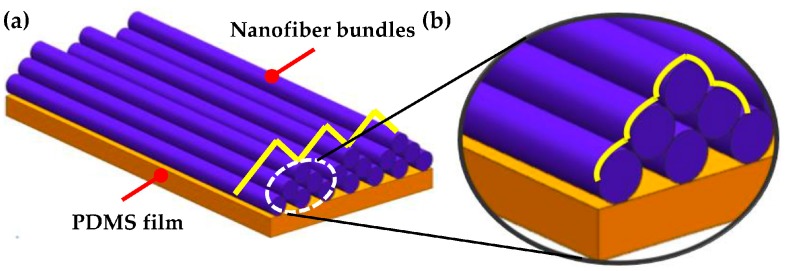
The surface morphology of uniaxially aligned nanofibers on a polydimethylsiloxane (PDMS) film: (**a**) bundles of nanofibers show the “groove” array, (**b**) each bundle shows “mini groove” array.

Here, the specific nanofiber bundles with customized patterns are investigated. The nanofiber collectors are designed and grounded for assembling electrospun polymeric nanofibers with specific patterns. As the cell culture substrate, the nanofiber bundles adhere tightly to the polydimethylsiloxane (PDMS) film. We conceived a semi-cured method in which the spin-coated PDMS film is not completely solidified before collecting the nanofibers. Nanofiber bundles are directly collected on a viscous PDMS film using the semi-cured method. As schematically shown in Figure 1, there are two types of “groove” arrays in the nanofiber bundle structure: one is between adjacent bundles and the other is among a single bundle. We designed and fabricated biocompatible polyvinylidene difluoride (PVDF) nanofiber bundles using the electrospinning technique to guide contractions of cardiac cell sheets.

## 3. Experimental Section

### 3.1. Nanofiber Preparation

**Figure 2:**
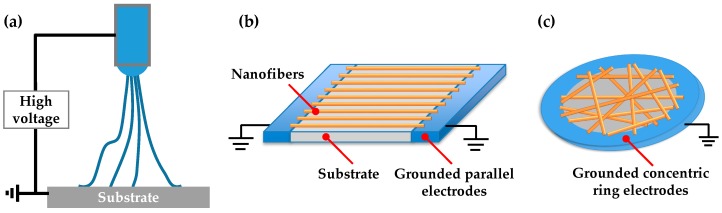
Design and fabrication of bundles of PVDF nanofibers with customized patterns: (**a**) schematic of the electrospinning process; (**b**) a parallel electrode pair is used for collecting uniaxially-aligned bundles of nanofibers on the PDMS film; and (**c**) a concentric ring electrode is used for collecting randomly-aligned bundles of nanofibers on the PDMS film.

PDMS (Sylgard 184) elastomer was mixed at the ratio of 10:1 (base to curing agent) and spin-coated on the glass cover slip at 3000 RPM for 30 s. The PDMS film was partially solidified at 60 °C for 3 h. The PVDF solution was prepared for electrospinning. PVDF pellets (molecular weight (M_w_) = 534,000 g·mol^−1^), *N*,*N*-dimethylacetamide (DMAC) and acetone were purchased from Sigma-Aldrich and were used as received. Solutions of 1.6 g PVDF were added to 10 mL DMAC/acetone solvent mixture (4/6 *v*/*v*), and then the mixture was stirred with a magnetic stirring bar at 60 °C for 4 h. The polymer solution was placed in a 1-mL plastic syringe tipped with a 25-gauge flat mouth stainless steel dispensing needle. A syringe pump was used to inject the polymer solution into the needle at a constant rate of 0.5 mL·h^−1^. Electrospinning was switched on when a 30 kV high voltage power supply was applied into the needle, as shown in Figure 2a. Specific electrodes were designed to pattern nanofibers on the semi-cured PDMS film; for example, a pair of parallel electrodes was used for uniaxial alignment and concentric ring electrode was used for a random straight pattern, as shown in Figure 2b,c. The distance was 12 cm from the needle to the PDMS film. The as-electrospun PVDF nanofibers settled on the semi-cured PDMS film and across the electrodes. Finally, the nanofibers were heated at 45 °C overnight to cause the retained solvents to evaporate.

### 3.2. Nanofiber Surface Functionalization

Before the cardiomyocytes were cultured on the nanofibers, the surface of the nanofibers and the PDMS film needed to be modified. The adhesive protein fibronectin (No. F0895, Sigma-Aldrich, St. Louis, MO, USA) was used to form chemical bonds with the PVDF chains and elicit cell adhesion and growth on the PVDF nanofibers. Immediately prior to fibronectin treatment, the nanofibers/PDMS film substrates were oxidized using ultraviolet (UV) ozone for at least 8 h to sterilize and increase the hydrophilicity of the surface. Then, fibronectin was deposited by placing a 2-mL droplet of 50 μg/mL fibronectin in sterile Hank’s balanced salt solution (HBSS) on the nanofibers/PDMS film for 30 min. Next, the extra fibronectin was removed by washing three times with HBSS and the film was then air dried.

### 3.3. Cell Isolation and Cell Culture

Cardiomyocytes were isolated from the ventricles of two-day-old Sprague-Dawley rats. All procedures were conducted according to the guidelines of the Institutional Animal Care and Use Committee (IACUC) at Tsinghua University. Firstly, ventricles were surgically isolated and digested in 0.1% trypsin solution overnight at 4 °C. After the supernatant was discarded, the second digestion was conducted by adding another digest enzyme (0.1% (*v*/*v*) collagenase II in HBSS) in a 37 °C water bath and stirring with a stir bar for 10 min at 100 RPM. This step was repeated around ten times, until the ventricle tissues disappeared. Subsequently, the isolated cardiomyocytes and fibroblasts were collected and re-suspended in the culture medium of 89% DMEM, 10% (*v*/*v*) heat-inactivated fetal bovine serum (FBS), and 1% (*v*/*v*) Penicillin/Streptomycin. The cells were allowed to adhere to the culture dish for a period of 1 h twice, and thus after the supernatant was discarded, the cardiomyocytes were left as the remains. Next, the cardiomyocytes were evenly seeded on the nanofibers/PDMS film at a density of 2.0 million cells, and incubated under standard conditions (37 °C and 5% CO_2_). Every 24 h in the incubation period, the cell culture substrates were washed three times with the culture medium to remove non-adherent cells, and they were then refreshed with new culture medium. 

### 3.4. Cellular Characterization

#### 3.4.1. Environmental Scanning Electron Microscopy

The morphology of cardiomyocytes and nanofibers was visualized by a scanning electron microscope (SEM) in a water environment (Quanta 450, FEI Company, Eindhoven, The Netherlands). The samples were prepared as follows. The cardiomyocytes were fixed with 2.5% glutaraldehyde in deionized water for 2 h and dehydrated with a series of graded ethanol (30%, 50%, 70%, 80%, 90% and 100%). Subsequently, the samples were immersed in tert-butyl alcohol twice for three minutes and then frozen at −20 °C until use. 

#### 3.4.2. Immunofluorescence Staining and Imaging

Samples were stained for the immunofluorescence imaging. The cardiomyocytes on nanofibers were washed three times with HBSS and fixed in 4% paraformaldehyde for 20 min and 0.5% TritonX-100 in HBBS for another 20 min at room temperature. The samples were blocked with 5% (*w*/*v*) bovine serum albumin (BSA, Wissent, Saint-Jean-Baptiste, QC, Canada) for another 20 min. Subsequently, the blocked samples were first stained using 1:200 dilutions of mouse anti-sarcomeric α-actinin monoclonal primary antibody, and then using goat anti-rabbit conjugated to tetramethylrhodamine secondary antibodies and 1:200 dilutions of DAPI, phalloidin conjugated to Alexa-Fluor 488 (Invitrogen, Carlsbad, CA, USA). After staining, the extra protein was removed with phosphate buffer saline (PBS) and the stained cardiomyocytes were mounted to glass slides and ready for imaging using a confocal microscope (AXIOobserze.Z1, Zeiss, Oberkochen, Germany).

#### 3.4.3. Atomic Force Microscopy Characterization

The contraction activity of the cardiomyocytes was studied using atomic force microscopy (AFM, NanoWizard^®^, JPK Instrument, Germany). The AFM tip used here was a silicon nitride microcantilever (nominal spring coefficient of 0.2 N/m; MLCT, Veeco, Plainview, NY, USA). The microcantilever tip was brought into gentle contact with one of the cardiomyocytes. The cardiomyocytes typically were maintained at a temperature of 37 °C. The force exerted on the cardiomyocyte by the tip was kept constant. The height of the tip varied with the cardiomyocyte contracting. Therefore, the real-time movement of the cell membrane was recorded precisely with the vertical displacement of the tip. Accordingly, the amplitude determined under the measurement represents the contractility or the contractile strength of the cardiomyocytes.

## 4. Results and Discussion

### 4.1. Pattern of Nanofiber Bundles

**Figure 3:**
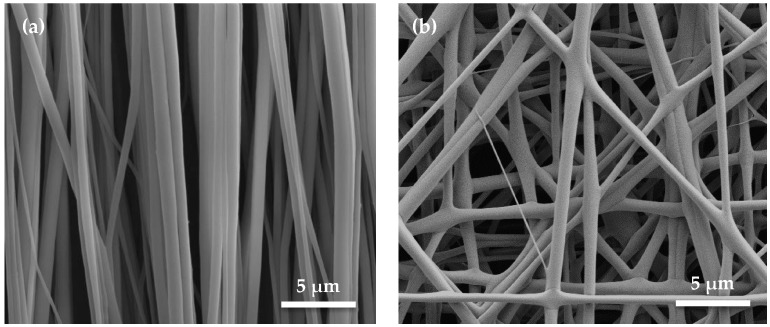
SEM images of (**a**) uniaxially aligned PVDF nanofibers and (**b**) randomly distributed PVDF nanofibers.

The morphology of the electrospun PVDF nanofibers was characterized using a field emission scanning electron microscope (SEM) (QUANTA FEG 450, FEI Company). As shown in Figure 3a, the PVDF nanofibers are aligned vertically and have very smooth surfaces. Around 10 nanofibers assemble into a bundle, which shows a groove structure between the bundles. So, the PVDF nanofiber bundles, considered as the cell culture substrate, can provide a 3D morphology. As a control, Figure 3b shows the randomly attributed nanofibers. There is a lot of void space between these nanofibers. Both the samples present a reliable interaction of all the nanofibers. Thus, the nanofibers can act as a robust substrate in the culture medium. 

### 4.2. Microstructures of 2D Cardiac Cell Sheets

**Figure 4:**
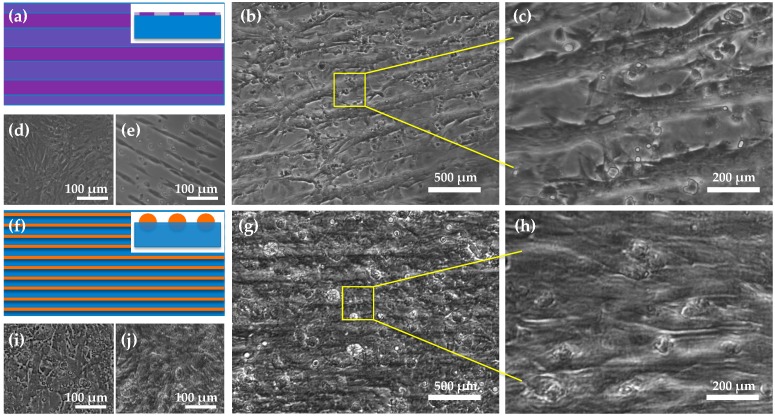
(**a**) Stripe pattern with alternating high- and low-density fibronectin lines (the inset shows the cross-section of the protein pattern on the PDMS thin film). (**b**,**c**) Anisotropic 2D myocardium was produced by culturing on the alternating high- and low-density fibronectin lines. (**d**) Isotropic 2D myocardium with uniform fibronectin coating, and (**e**) discrete cell lines cultured based on alternating fibronectin and non-fibronectin lines. (**f**) Electrospun PVDF nanofibers are aligned on/in PDMS thin film (the cross-section inserted shows that the nanofibers are partially embedded into the flexible PDMS film). (**g**,**h**) Cardiomyocytes grew on the nanofibers and were oriented along the alignment of the nanofibers. (**i**) Isotropic 2D myocardium with uniform fibronectin coating only on the PDMS film, and (**j**) Isotropic 3D myocardium growing on the nanofiber mat at random.

Figure 4 shows the microstructures of the 2D cardiac cell sheets on the planer PDMS film and the PVDF nanofibers. As shown in Figure 4a–c, the cardiomyocytes on the PDMS film substrate with the alternating high- and low-density fibronectin lines grow and align along the same direction. Moreover, all the cardiomyocytes contract at the same frequency and pace. However, this pattern does not result in the 3D cell culture. Figure 4d,e show the other cell patterns on the planer PDMS film, as a control. Therefore, we engineered anisotropic cardiac cell sheets on fibronectin-coated nanofibers by passively seeding cardiomyocytes, as shown in Figure 4f–h. Figure 4g,h show that the cell sheets have the uniaxial alignment of the cell bodies. It is suggested that the anisotropic pattern of the cardiomyocytes is realized through mechanical and chemical interfaces between the cardiomyocytes and PVDF nanofibers. Figure 4i,j show the cardiomyocytes cultured on the random nanofibers, as a control.

**Figure 5:**
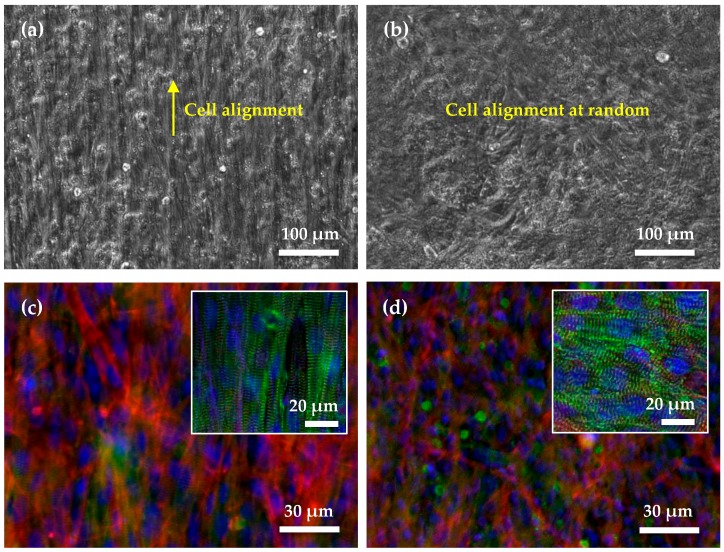
Microstructures of 2D cardiomyocyte sheets on bundles of PVDF nanofibers with uniaxial alignment (**a**,**c**) and random pattern (**b**,**d**), respectively. (**a**) Image of a cardiac cell sheet on uniaxially aligned nanofibers, (**b**) image of a cardiac cell sheet on random nanofibers, (**c**) immunofluorescence of nuclei (blue), F-actin (red) and sarcomeric a-actinin (green) of a cardiac cell sheet on uniaxially aligned nanofibers, and (**d**) immunofluorescence on random nanofibers. The insets of images (**c**,**d**) merge the α-actinin and nuclei images.

Here, the nanofibers serve as physical cues for the inter- and intracellular organization of cardiomyocytes into a tissue, and the uniaxial coupling of sarcomere ensembles over length scales from microns to centimeters. Compared with Figure 5b, the cell image in Figure 5a shows uniaxially aligned living cardiomyocytes on parallel nanofibers. The muscle strips therefore display functional behavior characteristics of physiological skeletal muscle. Staining for sarcomeric α-actinin (green), F-actin (red) and nuclei (blue) reveals uniaxial sarcomere alignment for the aligned pattern (Figure 5c), and no preferential alignment of sarcomeres along any axis for the random pattern (Figure 5d). The obvious shade in the two staining images implies the 3D structures of the cell sheets. For both kinds of nanofiber pattern, the nanofibers serve as physical cues for the accordingly patterned cardiomyocytes in a tissue. Therefore, synchronized actuation of the uniaxially aligned contractile cardiomyocytes is critical for unified functionality. 

### 4.3. Shape of Single Cell

**Figure 6:**
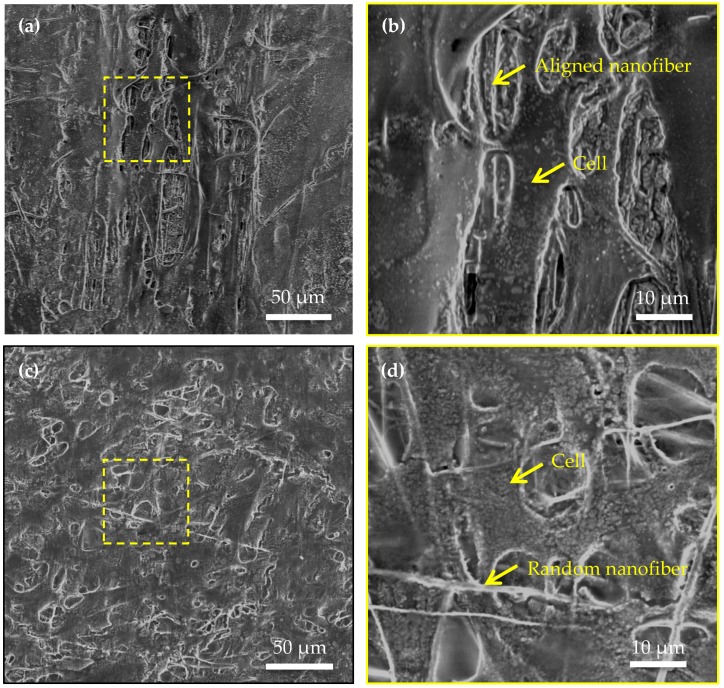
Environmental SEM images of cardiomyocytes on PVDF nanofiber bundles: (**a**) uniaxially aligned cardiomyocytes grow and adhere to the aligned nanofiber bundles, (**b**) the zoomed-in image of (**a**), (**c**) randomly distributed cardiomyocytes on random nanofiber bundles, and (**d**) the zoomed-in image of (**c**).

Figure 6 demonstrates the environmental SEM images of the interface of cardiomyocytes and nanofibers. Uniaxially aligned cardiomyocytes grew and adhered to the aligned nanofiber bundles (Figure 6a), which shows the interface of a single cardiomyocyte and parallel nanofibers, as shown in Figure 6b. Randomly distributed cardiomyocytes on random nanofiber bundles (Figure 6c) show the shape of the cardiomyocyte and the cell/nanofiber interface. The cardiomyocyte on the random nanofiber bundles has a pentagonal shape, as shown in Figure 6d.

### 4.4. Contraction of Cell Sheet

**Figure 7:**
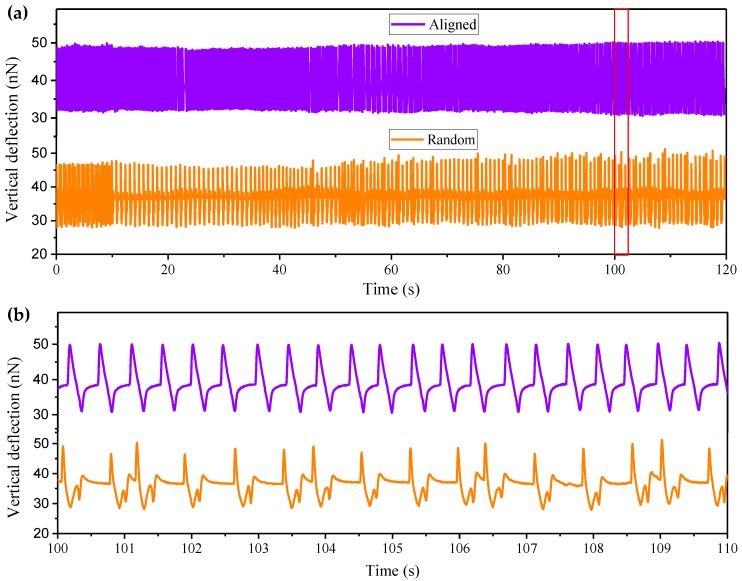
(**a**) Vertical deflection of one of the cardiomyocytes in the cardiac cell sheet is recorded for 2 min. The purple curve and orange curve represents the real-time contraction of the cardiomyocyte cultured on the uniaxially aligned nanofiber bundles and the random distributed nanofiber bundles, respectively. (**b**) An enlarged section of (**a**) is displayed to show the individual beats and periodicity of the contraction.

The mechanodynamic characteristics of the contraction of the cardiac cell sheet, like pulse vertical deflection and frequency, have been investigated using AFM [30]. When all the cardiomyocytes self-assembled into the confluent cell sheet on the uniaxial aligned nanofibers (as seen in the inset image of Figure 7a), a microcantilever tip was brought into gentle contact with the center of one of the cardiomyocytes to allow for patch-clamp recording [31]. Figure 7a shows that the real-time vertical deflection of the selected cardiomyocyte was tracked precisely with the vertical displacement of the microcantilever tip. The longtime vertical deflection of the cardiomyocyte on the uniaxially aligned nanofiber bundles shows a stable maximal value. The cardiomyocyte contracted periodically at a stable frequency of a 2.5 Hz, as shown in Figure 7b. As a control, the randomly distributed cardiomyocyte contracted in an unstable period, as shown in Figure 7a. The vertical deflection of the cell varied, as seen in the short-term recording of Figure 7b. The characterization and exploitation of such contraction functionality may facilitate the development of more biological machines.

## 5. Conclusions

To summarize, we demonstrated a new 3D cell culture substrate that is built by electrospun polymeric nanofiber bundles. This substrate can guide the cardiac cell orientating along the nanofiber bundles. By designing different patterns of the nanofibers, the interfacial structure shows that the cardiomyocytes interact with the nanofiber at the protein level and the cellular level, as shown by immunofluorescence imaging and ESEM imaging. The pattern of the nanofibers can influence the specific contraction of the cardiomyocytes as well. These results show a new method of cell alignment and an alternative approach to observe the cell/material interface.

## References

[1] Zuppinger C. (2015). 3D culture for cardiac cells. Biochim. Biophys. Acta.

[2] Battista S., Guarnieri D., Borselli C., Zeppetelli S., Borzacchiello A., Mayol L., Gerbasio D., Keene D.R., Ambrosio L., Netti P.A. (2005). The effect of matrix composition of 3D constructs on embryonic stem cell differentiation. Biomaterials.

[3] Ikonen L., Kerkelä E., Metselaar G., Stuart M.C., de Jong M.R., Aaltosetälä K. (2013). 2D and 3D self-assembling nanofiber hydrogels for cardiomyocyte culture. BioMed Res. Int..

[4] Emmert M.Y., Hitchcock R.W., Hoerstrup S.P. (2014). Cell therapy, 3D culture systems and tissue engineering for cardiac regeneration. Adv. Drug Deliv. Rev..

[5] Lee J.M., Mhawechfauceglia P., Lee N., Parsanian L.C., Lin Y.G., Gayther S.A., Lawrenson K. (2013). A three-dimensional microenvironment alters protein expression and chemosensitivity of epithelial ovarian cancer cells in vitro. Lab. Investig..

[6] Li Z., Guan J. (2011). Hydrogels for cardiac tissue engineering. Polymers.

[7] Khaoustov V.I., Darlington G.J., Soriano H.E., Krishnan B., Risin D., Pellis N.R., Yoffe B. (1999). Induction of threedimensional assembly of human liver cells by simulated microgravity. In Vitro Cell. Dev. Biol. Anim..

[8] Noto T., Tokuda Y., Nakamura Y., Suzuki A., Watanabe K., Yamamura M., Tajima T., Mitomi T., Nishijima K. (1989). A new high-yield continuous cell-culture system for lymphokine-activated killer cells. Cancer Immunol. Immunother..

[9] Barcellos-Hoff M.H., Aggeler J., Ram T.G., Bissell M.J. (1989). Functional differentiation and alveolar morphogenesis of primary mammary cultures on reconstituted basement membrane. Development.

[10] Hutmacher D.W. (2001). Scaffold design and fabrication technologies for engineering tissues—State of the art and future perspectives. J. Biomater. Sci. Polym. Ed..

[11] Lawrenson K., Benjamin E., Turmaine M., Jacobs I., Gayther S., Dafou D. (2009). In vitro three-dimensional modelling of human ovarian surface epithelial cells. Cell Prolif..

[12] Ghosh S., Spagnoli G.C., Martin I., Ploegert S., Demougin P., Heberer M., Reschner A. (2005). Three-dimensional culture of melanoma cells profoundly affects gene expression profile: A high density oligonucleotide array study. J. Cell. Physiol..

[13] Yeong W.Y., Sudarmadji N., Yu H.Y., Chua C.K., Leong K.F., Venkatraman S.S., Boey Y.C.F., Tan L.P. (2010). Porous polycaprolactone scaffold for cardiac tissue engineering fabricated by selective laser sintering. Acta Biomater..

[14] Yang S., Leong K.F., Du Z., Chua C.K. (2002). The design of scaffolds for use in tissue engineering. Part II. Rapid prototyping techniques. Tissue Eng..

[15] Lee J.M., Sing S.L., Tan E.Y.S., Yeong W.Y. (2016). Bioprinting in cardiovascular tissue engineering: A review. Int. J. Bioprint..

[16] Wang H., Vijayavenkataraman S., Wu Y., Shu Z., Sun J., Hsi J.F.Y. (2016). Investigation of process parameters of electrohydro-dynamic jetting for 3D printed PCL fibrous scaffolds with complex geometries. Int. J. Bioprint..

[17] Wang M., He J., Liu Y., Li M., Li D., Jin Z. (2015). The trend towards in vivo bioprinting. Int. J. Bioprint..

[18] Bhuthalingam R., Lim P.Q., Irvine S.A., Agrawal A., Mhaisalkar P.S., An J., Chua C.K., Venkatraman S. (2015). A novel 3D printing method for cell alignment and differentiation. Int. J. Bioprint..

[19] Jahnke H.G., Steel D., Fleischer S., Seidel D., Kurz R., Vinz S., Dahlenborg K., Sartipy P., Robitzki A.A. (2013). A novel 3D label-free monitoring system of hES-derived cardiomyocyte clusters: A step forward to in vitro cardiotoxicity testing. PLoS ONE.

[20] Zhao X., He J., Xu F., Liu Y., Li D. (2016). Electrohydrodynamic printing: A potential tool for high-resolution hydrogel/cell patterning. Virtual Phys. Prototyp..

[21] Tse C.C.W., Ng S.S., Stringer J., Macneil S., Haycock J.W., Smith P.J. (2016). Utilising inkjet printed paraffin wax for cell patterning applications. Int. J. Bioprint..

[22] Dan K., Prabhakaran M.P., Jin G., Ramakrishna S. (2011). Guided orientation of cardiomyocytes on electrospun aligned nanofibers for cardiac tissue engineering. J. Biomed. Mater. Res. Part B Appl. Biomater..

[23] Chanthakulchan A., Koomsap P., Parkhi A.A., Supaphol P. (2015). Environmental effects in fibre fabrication using electrospinning-based rapid prototyping. Virtual Phys. Prototyp..

[24] Parajuli D., Koomsap P., Parkhi A.A., Supaphol P. (2016). Experimental investigation on process parameters of near-field deposition of electrospinning-based rapid prototyping. Virtual Phys. Prototyp..

[25] Liu X., Wang X., Li S., Lin L. Energy harvesting using uniaxially aligned cardiomyocytes. Proceedings of the 2014 IEEE 27th International Conference on Micro Electro Mechanical Systems (MEMS).

[26] Chen Z.G., Wang P. (2010). Electrospun collagen-chitosan nanofiber: A biomimetic extracellular matrix for endothelial cell and smooth muscle cell. Acta Biomater..

[27] Ghasemi-Mobarakeh L., Prabhakaran M.P., Morshed M., Nasr-Esfahani M.H., Ramakrishna S. (2009). Electrical stimulation of nerve cells using conductive nanofibrous scaffolds for nerve tissue engineering. Tissue Eng. Part A.

[28] Zhu Y., Cao Y., Pan J., Liu Y. (2010). Macro-alignment of electrospun fibers for vascular tissue engineering. J. Biomed. Mater. Res. Part B Appl. Biomater..

[29] Liu X., Zhao H., Lu Y., Li S., Lin L., Du Y., Wang X. (2016). In vitro cardiomyocyte-driven biogenerator based on aligned piezoelectric nanofibers. Nanoscale.

[30] Grosberg A., Kuo P.L., Guo C.L., Geisse N.A., Bray M.A., Adams W.J., Sheehy S.P., Parker K.K. (2011). Self-organization of muscle cell structure and function. PLoS Comput. Biol..

[31] Alford P.W., Feinberg A.W., Sheehy S.P., Parker K.K. (2010). Biohybrid thin films for measuring contractility in engineered cardiovascular muscle. Biomaterials.

